# The role of passive immunization in the age of SARS-CoV-2: an update

**DOI:** 10.1186/s40001-020-00414-5

**Published:** 2020-05-13

**Authors:** Johannes C. Fischer, Kurt Zänker, Martijn van Griensven, Marion Schneider, Detlef Kindgen-Milles, Wolfram Trudo Knoefel, Artur Lichtenberg, Balint Tamaskovics, Freddy Joel Djiepmo-Njanang, Wilfried Budach, Stefanie Corradini, Ute Ganswindt, Dieter Häussinger, Torsten Feldt, Hubert Schelzig, Hans Bojar, Matthias Peiper, Edwin Bölke, Jan Haussmann, Christiane Matuschek

**Affiliations:** 1 Institute for Transplant Diagnostics and Cell Therapeutics, Heinrich Heine University, Düsseldorf, Germany; 2 The Nanjing Han & Zaenker Cancer Institute, Nanjing and Institute of Materia Medica, Chinese Academy of Medical Sciences & Peking Union Medical College, Nanjing Tech University, Jiangsu, China; 3 MERLN Institute for Technology-Inspired Regenerative Medicine, Department cBITE, Maastricht University, Maastricht, The Netherlands; 4 Department of Experimental Anesthesiology, University of Ulm, Ulm, Germany; 5 Department of Anesthesiology and Intensive Care Medicine, Heinrich Heine University, Düsseldorf, Germany; 6 Department of Surgery, Heinrich Heine University, Düsseldorf, Germany; 7 Department of Cardiac Surgery, Heinrich Heine University, Düsseldorf, Germany; 8 Department of Radiation Oncology, Heinrich Heine University, Moorenstr. 5, 40225 Düsseldorf, Germany; 9 Department of Radiation Oncology, University Hospital, LMU Munich, Munich, Germany; 10 Department of Radiation Oncology, Innsbruck, Austria; 11 Clinic of Gastroenterology, Hepatology und Infectious Diseases, Heinrich Heine University, Düsseldorf, Germany; 12 Department of Vascular Surgery, Heinrich Heine University, Düsseldorf, Germany; 13 NEXTGEN ONCOLOGY GROUP, Düsseldorf, Germany; 14 Heinrich-Heine-University, Düsseldorf, Germany

**Keywords:** Convalescent plasma, Covid-19, High risk, Bridge therapy, Intensive care unit, Respiratory failure, Immunology

## Abstract

The rapid spread of the corona virus pandemic is an existential problem for many people in numerous countries. So far, there is no effective vaccine protection or proven therapy available against the SARS-CoV-2 virus. In this review, we describe the role of passive immunization in times of the corona virus. Passive immunization could be a bridging technology to improve the immune defense of critically ill patients until better approaches with effective medications are available.

## Background

The continued occurrence of coronavirus epidemics/pandemics in certain periods poses a significant threat, in health, social, and economical terms. Ironically, even after a decade of research on coronavirus, there are still no licensed vaccines or therapeutic agents. In early January 2020, Robert L. Kruse advocated therapeutic strategies in an outbreak scenario to treat the novel coronavirus originating from Wuhan, China [1]. He recommended to start with new options, derived from and based on the knowledge of immunology, to fight the SARS-CoV-2 (severe acute respiratory syndrome coronavirus 2) and to treat patients under compassionate use, while formal clinical trials are conducted. This review takes this as its starting point and reviews the use of convalescent plasma as a potential therapy for COVID-19 as summarized, e.g., by Chen L et al. [2].

Passive immunotherapy is a very old procedure. The immunologist Emil von Behring introduced passive immunization in 1890 by developing a cure for diphtheria and tetanus using antibodies isolated from horse blood. Von Behring received the Nobel Prize for physiology and medicine in 1901 for his work. This approach was successfully used in other major epidemics, such as the Spanish flu in 1918, the measles epidemic in 1934 in the United States, more recently during the Middle East MERS epidemic in 2012, and against Ebola in 2015. This review describes the recent data concerning passive immunization against SARS-CoV-2 and whether this method could serve as a promising therapy option for patients until effective drugs or a vaccine are available.

Immunology clearly proves that antibodies in the blood or in the plasma fraction of the blood recognize epitopes on pathogens (e.g., viruses). They either neutralize them or reduce the virus load in conjunction with cellular responses to prevent or eventually cure the disease—thus antibodies are very efficient endogenous molecules that initiate and carry out self-healing processes in the human body.

Since more than 100 years, the history of medicine has now shown that the extraction and use of antibodies is an established principle for an immunological approach to combat pathogens. Transferring specific antibodies directed against certain disease-antigens to infected people as a passive immunization is an established therapeutic concept for many diseases like diphtheria, rabies, tetanus, and Ebola virus.

Despite the enormous advances in molecular biology, biotechnology, surgery and many other scientific areas, there is certainly nothing wrong with learning from past successes and adapting experiences to the problems of modern times, especially in the current pandemic Sars-CoV-2 crisis. Of note, physicians already observed healing in their patients using passive immunization as early as 1901, but antibodies and the underlying immunological concepts were not known then.

SARS-CoV-2 is a new pathogen to which humans of all ages have no immunity and are generally susceptible to infection. The virus is a new coronavirus that poses a global threat and imposes unprecedented burdens on healthcare providers and the healthcare system. Up to now, there is no specific vaccine or effective antiviral therapy against COVID-19 disease. From an immunological point of view, collected IgM and IgG antibodies from patients who have recovered from COVID-19, increase the chance to obtain neutralizing antibodies (NAbs) as therapeutic against the virus. Passive immunization is an alternative treatment strategy and could here experience a clinical renaissance until vaccines or antiviral medications become available. Especially for high-risk groups like very old people or cancer patients it could be a therapeutic option. New technologies and test systems enable us now to understand better the mechanisms in comparison to 1901, which finally can cure our patients.

### Passive immunization

The scientific evidence for passive immunization is generally overwhelming; but right now, in the therapeutic crisis of COVID-19, the renaissance of passive immunization is gaining scientific evidence. PubMed alone demonstrates an excessive growth in publications regarding this topic.

In a time of crisis, in which a therapy against a pathogen does not yet exist but this pathogen has triggered a pandemic crisis, every justifiable and documentable attempt must be made in order to achieve a therapeutic success with more than a low probability. This approach has now started in China and South Korea and case reports are available [3]. Meanwhile there a several publications, which show that the renaissance of passive immunization could be a bridging technology until effective medications or an active immunization is available. [2–10].

Shen C. and co-workers from China were the first who reported that convalescent plasma could be a treatment option for COVID-19 patients with respiratory failure. They reported their success with this approach in JAMA earlier this year [2]. The passive immunization improved the clinical situation in 5 patients where antiviral drugs or steroids were not effective. Patient’s viral loads decreased and became negative within 12 days after the transfusion [2]. One problem of this report is that all patients received antiviral medications and steroids before receiving their convalescent plasma. The latter treatment was performed as compassionate use as no other treatment therapy worked. Therefore, their results are very difficult to interpret. A few days later after this initial report, a case report from Korea showed that 2 elderly patients improved after the application of convalescent plasma. They published their results in a Korean journal [11]. One of the two patients was a 71-year-old man with no underlying medical conditions who was initially treated with malaria drugs and needed respiratory support for severe pneumonia. His condition improved when he was treated with convalescent plasma of a patient in his 20 s, along with steroids. The second patient, a 67-year-old female, did not respond to initial treatments including chloroquine, remdesivir, and oxygen therapy. She began to recover after receiving plasma therapy and steroids at the same time.

The largest report on patients treated with convalescent plasma is also from China [5]. Duan et al. reported their results in the *Proceedings of the National Academy of Sciences of the United States of America*. They enrolled prospectively 10 seriously ill patients with confirmed Sars-CoV-2 infection by real-time PCR in a clinical trial. A dose of 200 mL non-cryopreserved convalescent plasma derived from recently recovered patients with specific neutralizing antibody titers above 1:640 was transfused in addition to maximal supportive care and antiviral agents. The primary endpoint was the safety of convalescent plasma transfusion. Their second clinical endpoints were the improvement of clinical symptoms and normalization of laboratory parameters within 3 days after convalescent plasma transfusion. Their prospective trial showed that convalescent plasma therapy was well tolerated and could potentially improve the clinical outcomes through neutralizing viremia in severe COVID-19 cases.

A very recent report from Wuhan enrolled 6 COVID-19 patients in their case study, receiving 1–3 convalescent plasma infusions at d32 to d59 after onset of symptoms. In this case study, the convalescent plasma infusions were well tolerated also. Five patients with radiological lung finding (4 oxygenized) recovered and were PCR negative or discharged after 4–10 days after plasma infusion [10]. Interestingly in the 6th patient with only mild symptoms for more than 30 days the positive PCR throat swab findings could be cleared after plasma infusion.

Meanwhile, the FDA has approved convalescent plasma as a treatment option for critically ill COVID-19 patients [8]. The optimal dose and time point of application, as well as the clinical benefit of convalescent therapy, needs further investigation in larger well-controlled trials. Therefore, prospective randomized trials are planned in Germany and in the United States to answer the question whether convalescent plasma could serve as a treatment option for COVID-19 patients.

Passive immunization is an alternative treatment strategy and is now experiencing a clinical renaissance until vaccines or antiviral medications become available. Especially for high-risk groups such as very old people or cancer patients it could be a therapeutic option.

### SARS-CoV-2 immunology

**Fig. 1:**
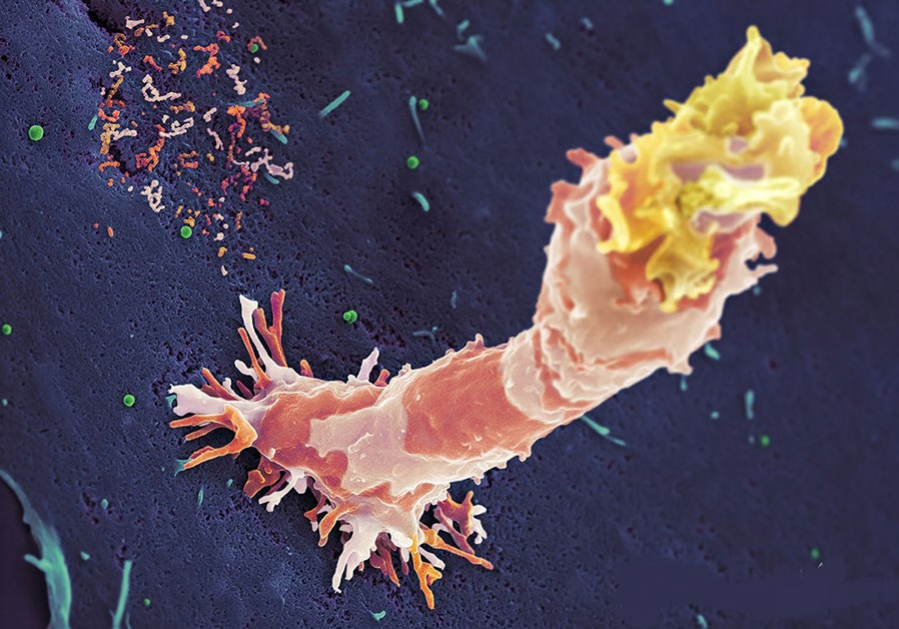
High activity natural killer cell during target attack. Footprint of previously attached natural killer cell can be identified by patchy membrane residuals on the target cell surface. Green objects have the size of budding virus particles. When will NK-cells join the cellular immune cascade to fight SARS-CoV-2?

In early January 2020, L.R. Kruse advocated therapeutic strategies in an outbreak scenario to treat the novel coronavirus originating from Wuhan, China [1]. The immune function is a strong and natural defense mechanism to fight against invasive pathogens (Fig. 1). However, it is necessary to recapitulate how the immune system works, considering the complexity of the interactions between the innate and adaptive immune system. In addition, the immune system needs some time to build up and specifically shape an armada of immunocompetent cells and humoral defense molecules—e.g., antibodies, pro-inflammatory and anti-inflammatory cytokines, complement system—to protect us individually from diseases. The innate immunity is the first line of defense from an evolutionary point of view. However, in contrast to an adaptive immune response, the receptors are strictly focused for limited reactions.

Recently, a case report was published reporting on the immune response of a COVID-19 patient with mild symptoms prior to recovery [12]. This study measured an increase of antibody-secreting cells (ASCs), follicular helper T cells (T_FH_ cells), activated CD4 + and CD8 + T cells and immunoglobulins IgM and IgG antibodies that bound to coronavirus SARS-CoV-2 before the symptoms diminished. This diversity of immune responses peaked in the peripheral blood around day 7 to day 9—concomitantly with the clearance of the virus and the ASCs and the T_FH_ cells were prominently present during convalescence (day 20). The authors also analyzed the co-expression of CD38 and HLA-DR, because these molecules are key elements for the activation of CD8-positive T-cells to fight against virus-infected cells. This part of the innate immune system increased also between days 7 and day 9 and declined again by day < 20 after infection [13] In addition, they monitored the CD16/CD14-positive cells in the peripheral blood and these pro-inflammatory, or alternatively called activated monocytes, showed a lower frequency, possibly indicating a migration of monocytes as phenotypical macrophages to the site of virus infection/virus load.

This case report shows very nicely that the immune system responds classically in a T cell-dependent manner prior to convalescence, because virus epitopes must have been presented within the HLA-DR of antigen-presenting cells (APC) (HLA class II) together with an appropriate pro-inflammatory cytokine profile that is regulated and segregated by helper T-cells. Furthermore, due to the appearance of activated CD8-positive cells, these cells must have been transformed into a cognate status by the HLA I presented virus-derived epitopes, which are expressed on infected soma cells (e.g., epithelial and alveolar cells of the respiratory tract); when cognate, these CD8-positive cells destroy and clear the virus-infected cells and thereby mitigate the virus load. Zhe Xu et al. published a case report describing the pathological findings in a COVID-19 patient with severe symptoms [14]. They also described—this is interesting to compare their report with that of a patient with mild symptoms—a hyperactive status of CD8-positive cells, which is evidenced by the proportions of HLA-DR and CD38 double-positive staining fractions. Moreover, there was an increased concentration of highly pro-inflammatory CCR6-positive and Th17-subpopulation in the CD4 T-helper cell fraction. The authors conclude that overactivation of adaptive T-cells, manifested by an increase of Th17-cells and highly cytotoxic CD8-positive T-cells accounts, at least in part, for the severe immune injury in this patient. It is noteworthy that both case reports did not describe that natural killer cells are partner within this cellular defense game. However, both patients did show exhausting T-cells, which was confirmed by Moon C [15].

It seems very likely that the mild, moderate or severe clinical course depends on the balanced interplay of the adaptive immunity by activation followed by a regular and timely shutdown of the key players. This is to ensure that no cytokine storm syndrome can develop that causes hyperinflammation [16]. This hyperinflammation is typically detrimental (comparable to a septic shock, post-traumatic organ dysfunction, etc.). Therefore, most anesthesiologists vote to screen COVID-19 patients for hyperinflammation to determine which patients may benefit from immunosuppression to prevent them from reaching a severe or fatal clinical status. Very recently, this hyperinflammation—likely caused by a chaotic, overwhelming cytokine profile—was confirmed by Vaninov. For patients experiencing a COVID-19 cytokine storm, the outcome is likely to be fatal [17].

There are four main subgroups of coronavirus, known as alpha, beta, gamma and delta. The virus is named for the crown-like spikes on its surface. It should be remembered that these spikes and all other viral structures represent different glycoproteins as epitopes for antigen-presenting cells (APC) (HLA class II), which are the important cells to initiate a classic immune response. If there are no correctly presented HLA class II peptide complexes presented by APC, there will be no initiation of an immune cascade by converting naïve immune cells into cognate ones.

The HLA signature is unique for each individual and therefore the presentation of epitopes from any invader is individual too—together with an individual danger signal [18]. Therefore, the way the immune system is activated is unique and depends on the signature and the invader [18]. It is more than likely that the different HLA-expression signatures within different ethnic groups in the world are the pillars how aggressively and sufficiently an individual organism attacks or even ignores an invader, like SARS-CoV-2.

What we call “inflammation” is an orchestrated process and an accumulation of cellular and humoral immune competence at the site of the invaders´ presence to eliminate the pathogen. Hereby, we consciously accept the risk of damaging the “inflamed” organ by hyperinflammation. This process of “hyperinflammation” might be triggered by the so-called damage-associated molecular patterns (DAMPs), which result from the invaders´ cellular decomposition (pathogen-associated molecular patterns (PAMPS) and trigger the inflammatory response of macrophages [19] Moreover, hyperinflammation may as well result from cytokine releasing, but killing-deficient natural killer cells recognizing virus-infected target, as described in children and animal models of hemophagocytic syndromes [20].

The different clinical outcomes of the SARS-CoV-2 infection with respect to morbidity and mortality may be explained by the different epitopes exposed by APC and the ethnic HLA signatures. As suggested by S.F. Bosten (personal communication) the severity of the infection in the USA may be due to the fact that some members of the SARS-CoV-2 subgroup are able to down-regulate HLA expression, and, therefore the affected patients are not protected anymore by building a sufficient immune competence against a virus-induced disease because of a pathogenic overload of the virus.

The immunological treatment of COVID-19 is still a puzzle with many missing pieces. It is evidence-based that the virus affects primarily T lymphocytes, which might result in decreased numbers, accompanied by a very enigmatic cytokine profile [21].

According to Cao X, a milestone regarding the implications for COVID-19 therapy is the acquisition of knowledge about the immunopathology of the virus–host interactions by measuring a logical plethora of biochemical and immunological parameters in order to arrange the puzzle pieces to complete the picture of understanding COVID-19.

The adaptive immune system is permanently able to “up-grade” its weapons to better respond to a similar attack at a later time and all the players are wired by the network of cytokines. However, the adaptive immune systems need time to learn. The learning process is mainly based on trial and error. Therefore, we, as human beings, have to accept to live with pathogenic invaders and only a high endemic infestation can protect us individually and epidemiologically from a personal or social crisis triggered by as yet not known pathogens/virus.

### Crosstalk of the immune system and the brain

Infection with SARS-CoV-2 apparently often goes hand in hand with neurological symptoms, which was described in a case series in *JAMA Neurology* mostly occurred at the beginning of the disease and were sometimes the reason for hospital admission. Smell and taste disorders are quite common [22].

We should not only consider the physical and medical aspects of the infection, but also the biopsychosocial implications caused by COVID-19. It is well established that there is an ongoing crosstalk between the brain and the organs initiated and permanently maintained by cytokines, which are released by immunocompetent cells as endocrine or paracrine (glial cells) messenger molecules at any time. The brain “knows” how the organs are functioning and is steering hereby the emotional status of a patient, e.g., in respect to depression, anxiety, fear, hope and self-esteem in healthy conditions [23], but also within the course of a disease by the so-called sickness behavior. The psychological/emotional status expressed as the patient´s mood in the course of a disease, favors or harms the process of healing as well—the immune system is with the cellular and humoral part an eminent conductor to orchestrate the patient’s well-being. The rapid transmission of the SARS-CoV-2 has emerged to mount serious challenges not only to hospitals including the critical units, but also the mental health service. Empathy and best medical care are equal partners when delivered by all caregivers to COVID-19 patients.

### Passive immunization for cancer patients and in solid organ transplantation

Meanwhile there are indications that the outcomes of COVID-19 infection in patients with cancer are more severe [24, 25]. In a retrospective cohort study from Wuhan, Zhang et al. reported in the *Annals of Oncology* the clinical characteristics and outcomes of COVID-19 patients. His team analyzed the infection in 28 patients with cancer from three hospitals in Wuhan, China. They found that these patients were at high risk for severe events with a higher mortality rate [26]. Cancer patients showed worsening health status and poor outcomes after COVID-19 infection. They recommend that cancer patients receiving anti-cancer treatments should have dynamic screening for COVID-19 infection and should avoid treatments causing immunosuppression or have their dosages reduced in case of COVID-19 co-infection. Accordingly, investigations have been initiated in Germany to analyze the incidence and prevalence of SARS-Cov-2 infections in cancer patients. One idea could be to conduct randomized trials and test if passive immunization is a valid treatment option for cancer patients before they undergo radio-chemotherapy or immunosuppressive chemotherapy. Especially leukemia patients have a high risk for poor clinical outcome upon CoV-2 infection [26]. Patients with solid organ transplantation would face the same dilemma. Organ-transplanted individuals require lifelong immunosuppression and are at risk to develop fatal COVID-19. Nevertheless, first observations have been published where organ-transplanted patients (liver and kidney) survived COVID-19 infection, though some problems such as rejection occurred. [27] There are currently no data available about a clinical benefit of passive immunization, though translation of current knowledge for organ transplant patients seems to favor for this treatment. This strategy seems to be even more important knowing that 72% of US centers have suspended live donor kidney transplantation and 68% live donor liver transplantation [28].

**Fig. 2:**
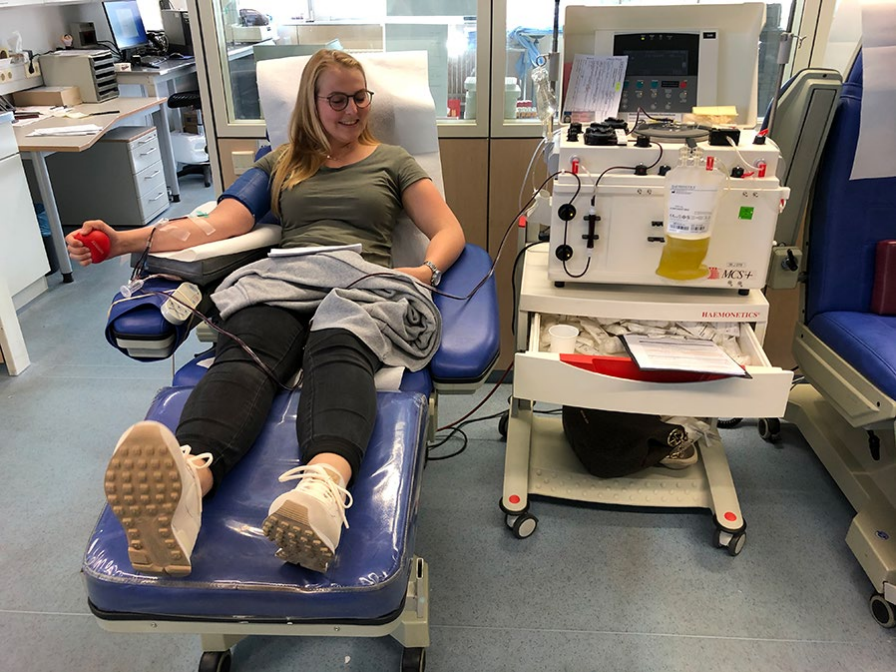
The first donor at the University of Dusseldorf on 16.04.2020. Three plasmas can be generated with one blood donation

Therefore, prospective randomized trials are necessary if passive immunization is a treatment option. Figure 2 depicts our first donor who spends her blood for one of these trials.

## Conclusion

The rapid spread of the corona virus pandemic is a life-threatening problem for many people in numerous countries. Passive immunization could be a bridging tool to improve the situation of critically ill patients until a better therapy with effective medications is available. In addition to the critically ill COVID-19 patients, prospective randomized trials are now planned to answer the question to whom this treatment option should be preferentially offered, prophylactically.

## Data Availability

All data and materials can be accessed via JF and EB.

## Abbreviations

APC: Antigen presenting cells
ASCs: Antibody-secreting cells
CD4: Cluster of differentiation 4
CD8: Cluster of differentiation 8
CD 14: Cluster of differentiation 14
CD 16: Cluster of differentiation 16
CD 38: Cluster of differentiation 38
COVID-19: Coronavirus disease 2019
DAMPs: Damage-associated molecular patterns e.g.exempli gratia
FDAUS: Food and drug adminstration
FH cells: Follicular helper cells
Flu: Influence
HLA-DR: Human leucocyte antigen DR isotype
IgG: Immunoglobulin G
IgM: Immunoglobulin M
JAMA: Journal of the American Medical Association
MERS: Middle East Respiratory Syndrome
Nabs: Neutralizing antibodies
NK: Natural killer cells
Proc Natl Acad Sci: Proceedings of the National Academy of Sciences of the United States of America
RNA: Ribonucleic acid
SARS-CoV-2: Severe acute respiratory syndrome coronavirus 2
