# Correction to: The role of passive immunization in the age of SARS-CoV-2: an update

**DOI:** 10.1186/s40001-020-00449-8

**Published:** 2020-10-30

**Authors:** Johannes C. Fischer, Kurt Zänker, Martijn van Griensven, Marion Schneider, Detlef Kindgen-Milles, Wolfram Trudo Knoefel, Artur Lichtenberg, Balint Tamaskovics, Freddy Joel Djiepmo-Njanang, Wilfried Budach, Stefanie Corradini, Ute Ganswindt, Dieter Häussinger, Torsten Feldt, Hubert Schelzig, Hans Bojar, Matthias Peiper, Edwin Bölke, Jan Haussmann, Christiane Matuschek

**Affiliations:** 1 Institute for Transplant Diagnostics and Cell Therapeutics, Heinrich Heine University, Düsseldorf, Germany; 2 The Nanjing Han & Zaenker Cancer Institute, Nanjing and Institute of Materia Medica, Chinese Academy of Medical Sciences & Peking Union Medical College, Nanjing Tech University, Jiangsu, China; 3 MERLN Institute for Technology-Inspired Regenerative Medicine, Department cBITE, Maastricht University, Maastricht, The Netherlands; 4 Department of Experimental Anesthesiology, University of Ulm, Ulm, Germany; 5 Department of Anesthesiology and Intensive Care Medicine, Heinrich Heine University, Düsseldorf, Germany; 6 Department of Surgery, Heinrich Heine University, Düsseldorf, Germany; 7 Department of Cardiac Surgery, Heinrich Heine University, Düsseldorf, Germany; 8 Department of Radiation Oncology, Heinrich Heine University, Moorenstr. 5, 40225 Düsseldorf, Germany; 9 Department of Radiation Oncology, University Hospital, LMU Munich, Munich, Germany; 10 Department of Radiation Oncology, Innsbruck, Austria; 11 Clinic of Gastroenterology, Hepatology und Infectious Diseases, Heinrich Heine University, Düsseldorf, Germany; 12 Department of Vascular Surgery, Heinrich Heine University, Düsseldorf, Germany; 13 NEXTGEN ONCOLOGY GROUP, Düsseldorf, Germany; 14 Heinrich-Heine-University, Düsseldorf, Germany

## Correction to: Eur J Med Res (2020) 25:16 10.1186/s40001-020-00414-5

Following publication of the original article [1], the authors identified an error in Fig. 1. The corrected Fig. 1 is given below.

**Fig. 1:**
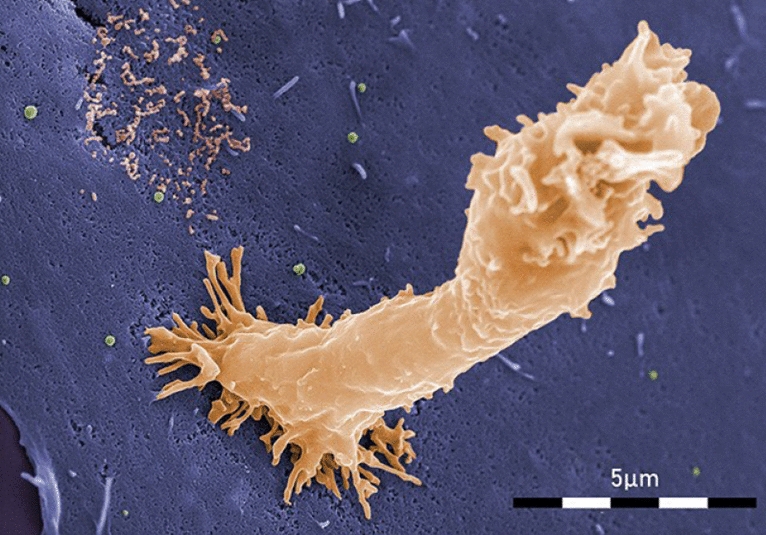
High activity natural killer cell during target attack. Footprint of previously attached natural killer cell can be identified by patchy membrane residuals on the target cell surface. Green objects have the size of budding virus particles. When will NK-cells join the cellular immune cascade to fight SARS-CoV-2?
